# Multidimensional performance assessment of public sector organisations using dominance criteria

**DOI:** 10.1002/hec.3554

**Published:** 2017-08-18

**Authors:** Nils Gutacker, Andrew Street

**Affiliations:** ^1^ Centre for Health Economics University of York York UK

**Keywords:** multidimensional, multilevel modelling, performance assessment, provider classification

## Abstract

Public sector organisations pursue multiple objectives and serve a number of stakeholders. But stakeholders are rarely explicit about the valuations they attach to different objectives, nor are these valuations likely to be identical. This complicates the assessment of their performance because no single set of weights can be chosen legitimately to aggregate outputs into unidimensional composite scores. We propose the use of dominance criteria in a multidimensional performance assessment framework to identify best practice and poor performance under relatively weak assumptions about stakeholders' preferences. We use as an example providers of hip replacement surgery in the English National Health Service and estimate multivariate multilevel models to study their performance in terms of length of stay, readmission rates, post‐operative patient‐reported health status and waiting time. We find substantial correlation between objectives and demonstrate that ignoring the correlation can lead to incorrect assessments of performance.

## 1 INTRODUCTION

When looking for service, consumers want the best and to avoid the worst. But telling these apart, and from service that lies between these extremes, is not always straightforward, particularly when it comes to evaluating services provided by organisations charged with serving the public. There are two key elements that complicate assessment of how well public sector organisations are doing their job (Besley & Ghatak, 2003; Dixit, 2002). First, they lack a single overarching objective against which performance can be assessed. Instead, they pursue multiple objectives, and this requires performance measurement along a range of performance dimensions. These objectives may conflict, such that higher performance along one dimension may come at the expense of performance along another. Second, they typically serve several stakeholders, including those using services, tax‐payers, regulatory bodies, and politicians. The values that stakeholders attach to objectives are often not known and unlikely to be identical (Devlin & Sussex, 2011; Propper & Wilson, 2012; Smith, 2002).

The lack of a set of common, explicit valuations for each performance dimension makes it difficult to construct a single, composite performance measure. The empirical literature has addressed this problem in different ways. Some performance evaluations restrict assessment to those dimensions for which explicit valuations have been expressed. Examples include Timbie, Newhouse, Rosenthal, and Normand (2008), Timbie and Normand (2008), and Karnon et al. (2013), which translate hospital mortality estimates into monetary units using the expressed valuation of a statistical life. The shortcoming of this approach is that performance dimensions that lack explicit valuations are omitted from the analysis.

Alternatively, analysts either choose a set of weights or implement pre‐defined scoring algorithms such as equal weighting, or derive weights from the data using approaches based on item response theory (Daniels & Normand, 2006; Landrum, Bronskill, & Normand, 2000; Landrum, Normand, & Rosenheck, 2003; Teixeira‐Pinto & Normand, 2008), data envelopment analysis (Dowd, Swenson, Kane, Parashuram, & Coulam, 2014), and ad hoc econometric specifications (Chua, Palangkaraya, & Yong, 2010). However, such practice conflicts with one of the key tenets of economic welfare theory, namely, that stakeholders are the best judges of their preferences (Boadway & Bruce, 1984; Smith & Street, 2005). There is no guarantee that weights imposed by analysts, however these are arrived at, match the preferences of all stakeholders. Consequently, those being assessed might legitimately question the validity of the generated composite score.

A growing literature in health economics has been concerned with multidimensional performance assessment of healthcare providers that avoids the need for a composite measure, see for example, Gutacker, Bojke, Daidone, Devlin, and Street (2013), Hall and Hamilton (2004), Hauck and Street (2006), Häkkinen et al. (2014), Kruse and Christensen (2013), Street, Gutacker, Bojke, Devlin, and Daidone (2014), Portrait, Galiën, and van den Berg (2016). This involves analysing performance against each dimension individually and then combining the results into a performance profile or balanced score card. In doing so, it makes explicit how healthcare providers perform on each performance dimension and how these dimensions correlate. However, the fundamental problem remains: performance profiles cannot be ranked, and so, it remains unclear which organisations excel or perform poorly across the board.

We seek to overcome this limitation by using dominance criteria to judge performance. Dominance criteria have been employed previously to assess ranking uncertainty associated with the weights used to construct a composite indicator of healthcare performance (Schang, Hynninen, Morton, & Salo, 2016), but, to the best of our knowledge, such criteria have not been applied when evaluating performance simultaneously across multiple dimensions. The concept of dominance is attractive in that it allows comparison of multidimensional performance profiles under relatively weak assumptions about stakeholders' utility functions. Indeed, the only requirement is that a judgement can be made about whether the marginal utility of an achievement is positive or negative and that this qualitative judgement applies to all stakeholders. We believe this to be a reasonable pre‐requisite in most contexts.

We illustrate our approach using data about providers of hip replacement surgery in the English National Health Service (NHS) during the period April 2009 to March 2012. Performance is assessed along four performance dimensions: inpatient length of stay (“efficiency”), waiting times (“access to care”), 28‐day readmission rates, and improvements in patient‐reported health status after surgery (both “clinical quality”), all of which have been the focus of recent health policy in England. We estimate multivariate multilevel models to account for the clustering of patients in hospitals and exploit the correlation of performance across dimensions (Zellner, 1962; Hauck & Street, 2006). Empirical Bayes estimates of the provider‐specific posterior means and variance–covariance matrices are used to classify hospitals into three categories: dominant, dominated, and non‐comparable.

The primary aim of this paper is to demonstrate how to apply dominance criteria to multidimensional performance assessment of public sector organisations. In the meeting this aim, we also demonstrate how to construct multivariate (rather than univariate) hypothesis tests of performance estimates that account for correlation between dimensions and thereby achieve correct coverage probabilities. Besides this, we make three further contributions to the empirical literature on hospital performance. First, we provide evidence about the correlations and thus the potential for trade‐offs, between different objectives that healthcare providers typically face. Previous research has focused predominantly on the association between hospital costs and mortality (see Hussey, Wertheimer, & Mehrotra, (2013), for a review), largely ignoring other important dimensions such as waiting times to access services or improvements in patients' health‐related quality of life. Second, in contrast to previous studies conducted at hospital level (e.g., Martin & Smith, 2005), we use individual‐level data and focus on a single homogeneous patient population, thereby reducing the risk of ecological fallacy. Third, by exploiting novel data on pre‐operative health status and by accounting for patients selecting into hospital, we are better able to identify the true impact that providers have on performance measures.

The remainder of this paper is structured as follows: In Section 2, we set out the assessment framework in conceptual terms. Section 3 presents the empirical methodology, and Section 4 describes our data. We report results in Section 5 and offer concluding comments in Section 6.

## 2 MULTIVARIATE PERFORMANCE ASSESSMENT USING DOMINANCE CRITERIA

Assume that a regulator, acting on behalf of stakeholders, seeks to determine the performance of a number of similar providers, such as hospitals, police forces, or schools. Let there be k=1,…,K performance dimensions with observed achievement Y_k_. Performance is determined by two factors, those under the control of the provider θ_k_ and external production constraints X_k_, so that

(1) $$Y_{k}=f(X_{k},\theta_{k})$$

for each provider.

The parameter θ_k_ can be interpreted as the provider's contribution to performance k over and above the circumstances in which they operate. This parameter is generally not directly observable and thus forms the target for inferences about performance within the framework of yardstick competition (Shleifer, 1985).

Each stakeholder derives utility from the performance of a provider so that U=U(θ_1_,…,θ_K_), which is assumed to be monotonic in θ_k_ over the range of realistic values for all k∈K. The regulator has only limited knowledge about the characteristics of this utility function. This may be because there are multiple stakeholders with heterogeneous and/or unknown preferences. More specifically, the regulator has no information about the marginal utility ∂ U/∂ θ_k_ that each stakeholder derives, and hence, the marginal rate of substitution (MRS) at which each stakeholder is willing to trade off performance on one dimension against that on another, that is, $MRS_{k,k^{\prime }}=\partial \theta_{k}/\partial \theta_{k^{\prime }}$ for $k\neq k^{\prime }$. However, the regulator has knowledge about the sign of ∂ U/∂ θ_k_, that is, whether achievements are expressed positively or negatively. To simplify the exposition, we assume that performance can be expressed so that utility increases in θ_k_.

If only one performance dimension is assessed (K=1) or the MRS across multiple dimensions are known, then achievements can be expressed as unidimensional (composite) scores. The regulator can then conduct either a relative or absolute assessment of performance. The first involves ranking providers j∈J according to their relative achievement θ_j_, where $\theta_{j}>\theta_{j^{\prime }}$ implies $U(\theta_{j})>U(\theta_{j^{\prime }})$ for $j\neq j^{\prime }$. This will result in a complete and transitive ordering of providers, assuming no ties. One can then designate some providers as performing well or poorly based on their relative ranking, for example, whether they fall within a given percentile of the distribution (Goldstein & Spiegelhalter, 1996). Alternatively, providers can be classified based on θ_j_−θ ^∗^ being larger or smaller than zero, where θ ^∗^ denotes an absolute performance standard to which providers are compared, 1Note that, when no external standards are specified, performance standards are typically based on the relative performance of all organisations (Shleifer, 1985). Hence, a provider will be considered to perform well when observed performance is better than a reference value derived from all providers. In many cases, this reference value is simply the average across all providers, that is, $\theta^{*}=\frac{1}{J}\sum \theta_{j}$.this being the approach to assessing standardised mortality after surgery (National Clinical Audit Advisory Group, 2011; Spiegelhalter, 2005), train punctuality (NetworkRail, 2016), or minimum standards for pupil achievement (Department of Education, 2016).

When multiple performance dimensions are assessed (K⩾2) and the MRS are unknown, a complete and transitive ordering of providers is no longer guaranteed and relative assessments are unfeasible. As a result, it becomes impossible to identify providers that perform well or poorly in terms of stakeholders' aggregate utility. However, some combinations of performance levels may be strictly preferable (dominant) or inferior (dominated) to other combinations, leading to a partial ordering of provider. As an analogue to the Pareto dominance criteria, we can formalise the following general dominance classification rules 2Devlin, Parkin, and Browne (2010) propose the use of a similar classification system to compare EuroQol‐5D (EQ‐5D) health profiles over time without resorting to making strong assumptions about patients' preferences.:

A provider either

1. dominates the comparator if $\theta_{jk}⩾\theta_{j^{\prime }k}$ for all k∈K and $\theta_{jk}>\theta_{j^{\prime }k}$ for some k∈K or
2. is dominated by the comparator if $\theta_{jk}⩽\theta_{j^{\prime }k}$ for all k∈K and $\theta_{jk}<\theta_{j^{\prime }k}$ for some k∈K or
3. is non‐comparable to the comparator if $\theta_{jk}⩾\theta_{j^{\prime }k}$ for some k∈K and $\theta_{jk}⩽\theta_{j^{\prime }k}$ for the remaining k∈K,

where $j\neq j^{\prime }$ and $\theta_{j^{\prime }k}$ denotes the performance level of the comparator, which may be either relative to other providers or to an absolute performance standard θ ^∗^.

## 3 METHODOLOGY

### 3.1 Empirical approach

The aims of the empirical analysis are to obtain estimates of provider performances θ_jk_ and of their correlation across each of the K=1,…,4 performance dimensions and to classify providers according to the dominance classification set out in Section 2. We estimate multivariate multilevel models (MVMLMs) with achievement Y_ijk_ observed for individuals served i=1,…,n_j_ who are clustered in j providers j=1,…,J. Multilevel (i.e., random intercept) models have become a staple tool in the field of performance assessment and allow us to (a) adjust performance for differences across providers in the characteristics of those served (i.e., risk adjustment), (b) decompose unexplained variation in achievement into random (within‐provider) variation at individual level and systematic (between‐provider) variation at provider level, and (c) obtain more reliable (precision‐weighted or shrunken) estimates of performance (Goldstein, 1997).

The multivariate nature of the data is taken into account through correlated random terms at each level of the hierarchy (Hauck & Street, 2006; Zellner, 1962). Allowing for correlation across dimensions is beneficial for several reasons. First, we can construct multivariate hypothesis tests of parameters of interest that take into account the correlation between dimensions and achieve correct coverage probabilities. We discuss this in detail in Section 3.2. Second, we obtain more efficient estimates of relevant parameters if either the components of X_ijk_ differ across k or non‐identity link functions are employed for at least some of the regression equations. Finally, by utilising a maximum likelihood estimator, missing data for any particular performance domain can be assumed missing at random conditional on all modelled covariates and observed performance (Goldstein, 1986; Little & Rubin, 1987).

In this application, we consider four dimensions of performance of which two are continuous and two are binary variables. In order to ascertain the conditional normality of error terms as imposed by the MVN assumption, we apply appropriate transformations (e.g., logarithmic) for the continuous variables and specify probit models for the binary variables, considering these as the observed realisation of a latent truncated Gaussian variable.

The empirical model to be estimated is specified as

(2) $$Y_{ijk}^{*}=\alpha_{k}+X_{ijk}^{\prime }\beta_{k}+\theta_{jk}+\epsilon_{ijk}$$

with $Y_{ijk}^{*}=f(Y_{ijk})$ for k=1,2 and

$$Y_{ijk}=\left\{\begin{array}{c} 1\;\text{if}\;Y_{ijk}^{*}>0\\ 0\;\text{if}\;Y_{ijk}^{*}⩽0 \end{array}\right.$$

for k=3,4.

The variable Y_ijk_ denotes observed performance, $Y_{ijk}^{*}$ is the corresponding latent underlying variable, f(.) is a transformation function chosen to normalise the conditional distribution of ϵ_ijk_,X_ijk_ is a vector of explanatory variables whose components may differ across dimensions, α_k_ is an intercept term, θ_jk_ denotes a random effect at provider level, and ϵ_ijk_ denotes the random error term at individual level. Both random terms are assumed to be MVN distributed with mean vector zero and a K×K variance–covariance matrix, so that θ_jk_∼M V N(0,Σ) with

$$\begin{array}{ccc} E(\theta_{jk}) & =0 & \\ var(\theta_{jk}) & =\tau_{k}^{2}\\ cov(\theta_{jk},\theta_{jk^{\prime }}) & =\rho_{\theta }\tau_{k}\tau_{k^{\prime }} \end{array}$$

for all $k\neq k^{\prime }$.

And similarly ϵ_ijk_∼M V N(0,Ω) with

$$\begin{array}{ccc} E(\epsilon_{ijk}) & =0 & \\ var(\epsilon_{ijk}) & =\sigma_{k}^{2}\;\text{for}\;k=1,2\\ var(\epsilon_{ijk}) & =1\;\text{for}\;k=3,4\\ cov(\epsilon_{ijk},\epsilon_{ijk^{\prime }}) & =\rho_{\epsilon }\sigma_{k}\sigma_{k^{\prime }} \end{array}$$

for all $k\neq k^{\prime }$. The model reduces to a set of univariate models if all off‐diagonal elements of Σ and Ω are zero, that is, achievements are uncorrelated conditional on observed patient factors.

### 3.2 Classification of provider effects and multivariate hypothesis tests

We compare providers against a common performance standard, which can be specified externally or set at some point along the observed distribution of performance such as the top decile (Burgess, Christiansen, Michalak, & Morris, 2000). For illustrative purposes, we define the standard as the expected performance of a (hypothetical) provider of average performance α_k_, that is, the conditional mean. We base our assessment of provider performance on estimates of θ_jk_, which represent the provider‐specific deviation from this benchmark and can be obtained using Empirical Bayes predictions techniques (Skrondal & Rabe‐Hesketh, 2009). A provider's dominance classification is determined by comparing its estimated adjusted achievements to that of the performance standard across all performance dimensions simultaneously. This leads to three possible classifications: dominant, dominated, or non‐comparable.

**Figure 1:**
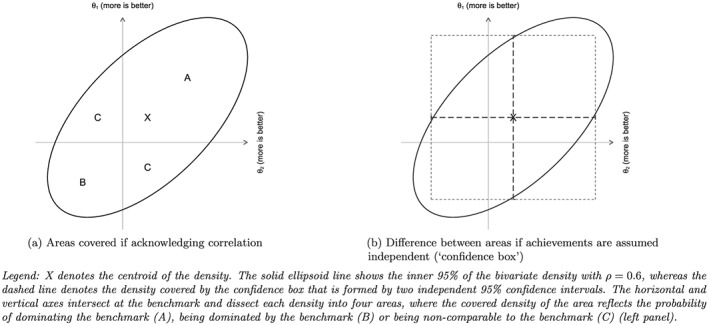
Example of area of probability density plane covered under different assumptions about the dependence of achievement scores

We quantify uncertainty around these possible classifications by taking a Bayesian perspective and calculating the posterior probability that a given provider truly dominates [is dominated; non‐comparable]. This involves calculating the area under the MVN probability density function that covers each of the three possibilities, for each provider. 3Our problem is similar to that encountered in the context of cost‐effectiveness analysis, where one wishes to compute the probability that a new treatment is cost‐effective for a given level of willingness to pay (Briggs and Fenn, 1998; O'Hagan et al., 2000; Van Hout, Al, Gordon, & Rutten, 1994).Figure 1a illustrates this for the two‐dimensional case with two highly correlated bivariate normal distributed achievements (ρ=0.6). The centroid of the density is given by X, and the ellipse shows the central 95% of this density. The density is dissected by two lines that intersect at the benchmark. The density covered by the Areas A and B equals the probability of dominating or being dominated by the benchmark, whereas the density covered by Area C gives the probability for the non‐comparable outcome. To calculate these probabilities, we follow the simulation approach of O'Hagan, Stevens, and Montmartin (2000). This involves drawing S repeated samples from the MVN posterior distribution of the provider‐specific Empirical Bayes estimates of the mean vector θ_j_ and associated variance–covariance matrix Σ_j_. We then apply the dominance criteria to each simulation and calculate posterior probabilities by averaging across simulations. Formally,

(3) $$Pr(\text{dominant}\;|\;J=j)=\frac{1}{S}\overset{S}{\underset{s=1}{\sum }}\overset{K}{\underset{k=1}{\prod }}I(\theta_{jk}^{s}>0)$$

(4) $$Pr(\text{dominated}\;|\;J=j)=\frac{1}{S}\overset{S}{\underset{s=1}{\sum }}\overset{K}{\underset{k=1}{\prod }}I(\theta_{jk}^{s}<0)$$

and by construction

(5) Pr(non‐comparable|J=j)=1−(Pr(dominant|J=j)+Pr(dominated|J=j))

where S is the total number of simulations, $\theta_{jk}^{s}$ denotes the simulated provider‐effect in simulation s, and I is an indicator function that takes the value of one if the condition is true and zero otherwise. This approach has several advantages over a series of univariate assessments. Most importantly, it accounts for the correlation between performance dimensions and thus achieves correct coverage of the confidence region (Briggs & Fenn, 1998). Figure 1b illustrates the difference between probability statements if performances on both dimensions are incorrectly assumed to be independent. The dashed line outlines the resulting “confidence box,” which is formed by the end points of two independent 95% confidence intervals that are adjusted for multiple testing. Furthermore, because we make probability statements about a single quantity of interest, the provider's location in the k‐dimensional performance space, we avoid such issues of multiple testing.

### 3.3 Risk‐adjustment

Perhaps the primary reason that observed achievements differ across hospitals is because they treat different types of patients. Accounting for these differences may not be necessary in contexts where funds have been allocated to achieve a standard level of outcome using some form of risk‐adjusted reimbursement formulae (Jacobs, Smith, & Street, 2006; Schang et al., 2016; Smith, 2003). However, in many healthcare systems, hospitals are subject to a prospective payment system that uses fairly crude payment categories to reimburse hospitals for differences in patients (Kobel, Thuilliez, Bellanger, & Pfeiffer, 2011). If the payment categories fail to account for all observable risks driving variation in outcomes, as in this context, risk‐adjustment will be necessary.

We perform risk‐adjustment for three of the four performance dimensions. No risk‐adjustment was performed in the analysis of waiting times because hospitals are expected to manage their waiting lists so as to balance high priority cases and those with less urgent need for admission (Gutacker, Siciliani, & Cookson, 2016). But for the other three dimensions, observed performance is likely to be related to patient characteristics. We constructed a “core” set of risk‐adjustment variables that are applied to all three dimensions and also considered additional characteristics for each dimension. Preliminary modelling of potential risk‐adjusters was conducted on the basis of univariate multilevel regression models and Locally Weighted Scatterplot Smoothing (LOWESS) plots. All continuous variables were first added linearly to the regression model, and we subsequently explored whether squared terms improved the fit of the model.

### 3.4 Endogeneity due to selection of provider

Patients in the English NHS have a right to choose their provider of inpatient care for most elective procedures. This may lead to endogeneity bias in the estimates of hospital performance if both the choice of hospital 4Selection may also arise if providers cream‐skim.and the level of service provided for an individual patient are driven by common underlying factors (e.g., unobserved severity and health literacy) that are not controlled for as part of *X*_*ijk*_(Geweke, Gowrisankaran, & Town, 2003; Gowrisankaran & Town, 1999).

In order to test for bias due to patient selection and to obtain correct estimates of hospital performance, we estimate the model in (2) and perform two‐stage residual inclusion as suggested by Terza, Basu, and Rathouz (2008). In the first stage, we estimate a multinomial model where choice of hospital is assumed to be determined by the straight‐line distance 5We also include distance^2^ and distance^3^ as well as an indicator for whether the hospital is the closest alternative. Hospitals with less than 30 patients were removed from the choice set. The patient's residence was approximated by the centroid of the lower super output area in which the patient lives. Lower super output areas are designed to include approximately 1,500 inhabitants, that is, they are substantially smaller than US ZIP codes.from the patient's residence to the hospital, an unobserved patient effect and random noise. Distance is commonly chosen as an instrumental variable as it is a major driver of hospital choice and is exogenously determined, on the reasonable assumption that patients do not choose where to live based on hospital performance (Gowrisankaran & Town, 1999). The residual from this regression captures both the unobserved patient effect and random noise. In the second stage, we enter this residual as an additional regressor into each of the four regression models. If the coefficients on the first‐stage residuals are statistically significantly different from zero, this provides evidence of selection bias and the need for two‐stage residual inclusion adjustments (Terza et al. 2008).

## 4 DATA

Our sources of data are the Hospital Episode Statistics (HES) and the Patient Reported Outcome Measures (PROM) survey. HES contains detailed inpatient records for all patients receiving NHS‐funded care in England. The PROM survey invites all patients undergoing unilateral hip replacement to report their health status before and 6 months after surgery using the Oxford Hip Score (OHS 6All patients are also invited to fill in the EQ‐5D questionnaire, a generic health‐related quality of life instrument (Brooks, 1996). However, we focus on the OHS as it is better approximated by a continuous distribution, and we do not seek to make comparisons across disease areas. Furthermore, the OHS is the relevant outcome measure for the newly introduced best practice tariff (a pay‐for‐performance scheme) in the English NHS that was introduced in April 2014 (Monitor and NHS England, 2013). Previous comparisons have demonstrated that performance assessments based on the EQ‐5D and OHS lead to similar conclusions (Neuburger, Hutchings, van der Meulen, & Black, 2013).; Dawson, Fitzpatrick, Carr, & Murray, 1996). The inpatient records are linked to survey responses using unique anonymised patient identifiers.

We extract information on all patients undergoing unilateral hip replacement (identified through the primary procedure code; Department of Health, 2008) in the period April 2009 to March 2012. 7Hospital Episode Statistics records activity at the level of “finished consultant episodes,” and we link consecutive episodes within the hospital stay and across hospital transfers to form continuous inpatient spells. A continuous inpatient spell is deemed complete when the patient is discharged from one provider and not re‐admitted to another provider within 2 days.Patients were excluded if they were aged 17 or younger at the time of admission, underwent revision surgery, or were admitted as emergencies or day cases. Patients were also excluded if they attended a provider that treated fewer than 30 patients in the same financial year.

For each patient, we extract information about demographic and medical characteristics, the admission process, and the hospital stay. The HES data are used to construct three performance measures: (a) inpatient length of stay (top‐coded at the 99th percentile), (b) emergency re‐admission within 28 days of discharge for any condition (coded as 0=not re‐admitted, 1=re‐admitted), and (c) waiting time, measured as the time elapsed between the surgeon's decision to admit and the actual admission to hospital. Waiting time is categorised into waits of no more than 18 weeks (=0) and waits exceeding 18 weeks (=1) to mirror the contemporaneous waiting time performance standard in the English NHS. 8The current performance standard is defined in terms of proportion of patients exceeding a waiting time of 18 weeks between the general practitioner's referral and the admission.


The fourth measure, post‐operative health status, is derived from the PROM survey and is based on the OHS. This is a reliable and validated measure of health status for hip replacement patients and consists of 12 questions regarding functioning and pain. For each item, the patient is asked to respond on a 5‐item scale. These items are summed to generate an index score ranging from 0 (*worst*) to 48 (*best*). Pre‐operative survey responses are collected by paper questionnaire during the last outpatient appointment or on the day of admission, whereas follow‐up responses are collected via mailed survey to the patient's home address. Participation in the PROM survey is voluntary for patients but mandatory for all hospitals providing NHS‐funded care to these patients. Approximately 60% of patients returned completed pre‐operative questionnaires that can be linked to their HES record (Gutacker, Street, Gomes, & Bojke, 2015). These patients tend to be slightly older, less likely to be male, and more likely to have been admitted as an emergency in the past year; see Table A1 for full descriptive statistics. Implicitly, we treat those observations to be missing completely at random, 9Hence, the probability of being included in our estimation sample is entirely random and determined neither by (un‐)observed patient or provider characteristics nor by the outcome of interest.which is in line with the official adjustment methodology of the national PROM programme. We return to this point in the discussion.

Based on previous research (Gutacker et al., 2016; Street et al., 2014), we identified a set of “core” patient characteristics that were included in all risk‐adjustment models: age, sex, primary diagnosis (coded as osteoarthritis [ICD‐10: M15‐19], rheumatoid arthritis [ICD‐10: M05‐06], or other), comorbidity burden as measured by individual Elixhauser comorbidity conditions recorded in secondary diagnosis fields (Elixhauser, Steiner, Harris, & Coffey, 1998), number of emergency admissions to hospital within the last year (coded as 0=none, 1=one or more), and patients' approximate socio‐economic status based on level of income deprivation in the patient's neighbourhood of residence as measured by the Index of Multiple Deprivation 2004 (Noble, Wright, Smith, & Dibben, 2006). The pre‐operative OHS score from the PROM survey is used to control for initial health status at admission. We also constructed other risk‐adjustment variables from the PROM survey, namely, the duration of problems, and whether the patient lives alone, considered herself disabled, or required help filling in the questionnaire. In the length of stay model, we controlled for the healthcare resource group (HRG, the English equivalent of Diagnosis‐Related Groups) to which the patient was allocated and which form the basis of the prospective payment system used to reimburse English hospitals (Grašič, Mason, & Street, 2015).

All continuous variables were mean centred to facilitate interpretation of the intercept. Our exploratory work confirmed the importance of all core variables in explaining variation in each of the three performance dimensions. Time with symptoms, assistance, and living alone did not explain variation in the probability of being re‐admitted and were thus not included in the final model for that dimension. Non‐linear effects were found for age (all performance dimensions) and pre‐treatment health status (only length of stay and post‐operative OHS).

## 5 RESULTS

### 5.1 Descriptive statistics

The estimation sample consists of 95,955 patients treated in 252 hospitals during April 2009 and March 2012. Table 1 presents descriptive statistics. Patients are on average 67 years old, and 41% of patients are male. The majority (68%) report having had problems with their hip joint for 1 to 5 years, with 8% experiencing symptoms for more than 10 years and 14% for less than 1 year, and 39% say they have a disability, and 27% live alone.

**Table 1:** Descriptive statistics

Description	N	Mean	SD
Achievement measures (dependent variables)
Post‐operative OHS	81,336	38.50	9.21
Length of stay (in days)	95,878	5.36	3.75
Waiting time > 18 weeks	92,154	0.17	0.38
28‐day emergency readmission	95,955	0.05	0.22
Patient characteristics (control variables)
Patient age (in years)	95,955	67.43	11.29
Patient gender (1 = male, 0 = female)	95,955	0.41	0.49
Pre‐operative OHS	95,955	17.66	8.28
Primary diagnosis			
Osteoarthritis	95,955	0.93	0.25
Rheumatoid arthritis	95,955	0.01	0.07
Other	95,955	0.06	0.24
Number of Elixhauser comorbidities			
0	95,955	0.35	0.48
1	95,955	0.29	0.45
2–3	95,955	0.26	0.44
4+	95,955	0.10	0.31
Previously admitted as an emergency (1 = yes, 0 = no)	95,955	0.08	0.28
Socio‐economic status	95,955	0.12	0.09
Disability (1 = yes, 0 = no)	95,955	0.39	0.49
Living alone (1 = yes, 0 = no)	95,955	0.27	0.44
Assistance (1 = yes, 0 = no)	95,955	0.21	0.41
Symptom duration			
< 1 year	95,955	0.14	0.35
1–5 years	95,955	0.68	0.47
6–10 years	95,955	0.11	0.31
> 10 years	95,955	0.08	0.26
Healthcare resource group
HB12C—Category 2 without CC	95,955	0.77	0.42
HB11C—Category 1 without CC	95,955	0.10	0.29
HB12B—Category 2 with CC	95,955	0.07	0.26
HB12A—Category 2 with major CC	95,955	0.04	0.19
HB11B—Category 1 with CC	95,955	0.01	0.11
other	95,955	0.02	0.12

*Abbreviation:* CC = complications or co‐morbidities; OHS = Oxford Hip Score; *N*= number of observations; *S* *D*= standard deviation.

*Note.* Healthcare resource groups refer to major hip procedures for non‐trauma patients in category 1 (HB12×) or category 2 (HB11×). Socio‐economic status is approximated by the % of neighbourhood residents claiming income benefits. This characteristic is measured at neighbourhood level (lower super output area).

**Figure 2:**
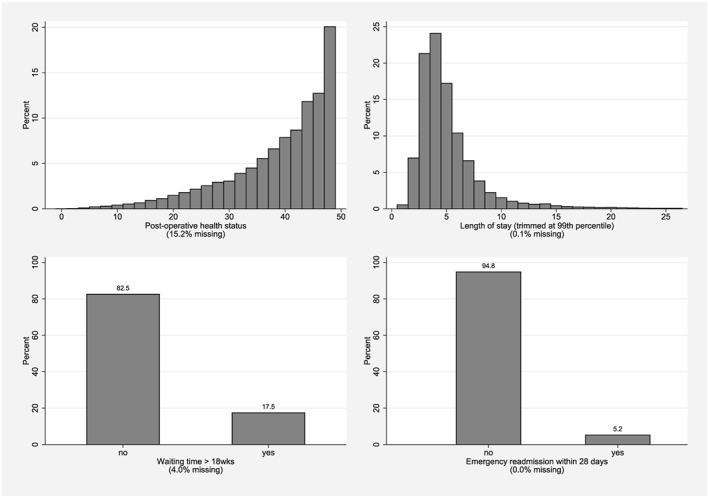
Empirical distribution of unadjusted achievement scores

Figure 2 illustrates the empirical distributions of the performance variables on their untransformed scales. The average post‐operative OHS is 38.5 (*S* *D*=9.2), and the average length of stay is 5.4 days (*S* *D*=3.8), with both distributions showing substantial skew. About 17.5% of patients waited longer than 18 weeks to be admitted to hospital, and 5.2% were readmitted to hospital within 28 days of discharge. There is a substantial proportion of missing responses in terms of post‐operative OHS (15.2%) and, to lesser degrees, waiting time (4.0%) and length of stay (0.1%). Conversely, emergency re‐admission status is recorded for all patients.

### 5.2 Hospital heterogeneity and correlation between performance dimensions

From the estimated variance–covariance matrices Σ and Ω, we can calculate the correlation across performance estimates. 10With the exception of waiting times, estimates are risk‐adjusted. The estimated coefficients on risk‐adjustment variables and associated standard errors are not the focus of this paper and are reported in Table A2 in the Appendix. The first‐stage residuals from the selection equation are jointly statistically significant (χ ^2^(4) = 14.97; p<.01) when entered into the main equations, suggesting that self‐selection into hospital may bias performance estimates if uncontrolled for (see Table A3 in the Appendix for first‐stage estimates).The lower off‐diagonal in Table 2 shows the correlation between performance estimates at provider level, whereas the upper off‐diagonal shows the correlation at patient level. Bold numbers indicate that the correlation coefficient is statistically significantly different from zero (p<.05; Huber–White standard errors).

**Table 2:** Correlation between performance dimensions

Performance dimension	(1)	(2)	(3)	(4)
Length of stay (1)	1.00	−**0.13**	**0.02**	**0.02**
Post‐operative OHS (2)	−**0.34**	1.00	−**0.02**	−**0.07**
Waiting time > 18 weeks (3)	**0.26**	−**0.31**	1.00	0.00
28‐day emergency readmission (4)	0.03	−**0.49**	0.16	1.00

*Note.* Lower triangle reports the correlation between random effects at provider level, whereas upper triangle (in italics) reports the correlation between random effects (i.e., the idiosyncratic error term) at patient level. Bold indicates that the correlation is statistically significantly different from zero at the 95% level.

There are significant correlations for four combinations of dimensions. Hospitals with shorter length of stay also realise better post‐operative health status for their patients (ρ = −0.34; S E=0.067; p<.001). This is consistent with findings from randomised controlled trials that tested the effectiveness of so‐called fast track or enhanced recovery pathways and found that hospitals that mobilise patients sooner after surgery were able to discharge them quicker and achieve better post‐operative outcomes (Husted, Holm, & Jacobsen, 2008; Larsen, Sørensen, Hansen, Thomsen, & Søballe, 2008).

Hospitals that have a lower proportion of patients waiting more than 18 weeks to be admitted also have a shorter length of stay (ρ= 0.26; S E=0.065; p<.001), suggesting better management of capacity and of their waiting lists. This would be consistent with a queuing model of limited bed capacity, where prospective patients cannot be admitted until current patients are discharged. Hospitals that have better post‐operative health outcomes also tend to have a lower proportion of patients waiting for more than 18 weeks (ρ= −0.31; S E=0.071; p<.001). Finally, the correlation between the probability of an emergency readmission within 28 days and post‐operative health status is negative and statistically significant (ρ = −0.49; S E=0.078; p<.001), which may indicate that readmission has an adverse impact on health status.

Overall, these correlations indicate that inferences based on a series of univariate assessments would likely be misleading and that our MVMLM is preferable for this empirical analysis of provider performance.

We have conducted sensitivity analyses with respect to two modelling choices (results are reported in Tables A4 and A5). First, we restricted analysis to public (NHS) hospitals and excluded private hospitals (so‐called independent sector treatment centres (ISTCs)) as these may operate under different production constraints. The estimated covariance terms in Σ are attenuated somewhat, and the correlations of waiting time with length of stay (p=.174) and post‐operative health status (p=.857) are no longer statistically significant.

Second, we included additional regressors based on patient risk factors averaged at hospital level to correct for potential bias 11This bias is likely to be small. We compared coefficient estimates from fixed and random effects estimators using Hausman tests and found little practical difference between those estimates, although the tests all rejected the assumption of unbiasedness for the random effects approach. This is likely to be due to our large sample, where within effects swamp between effects and the Hausman test is overpowered. Results are available from the authors on request. arising from correlation between X_ij_s and the hospital random effects (Mundlak, 1978). Due to convergence problems, we restricted these additional regressors to average patient age, pre‐operative PROM score, and level of income deprivation. Again, covariance terms are smaller in size but remain statistically significant.

### 5.3 Hospital performance assessment

**Figure 3:**
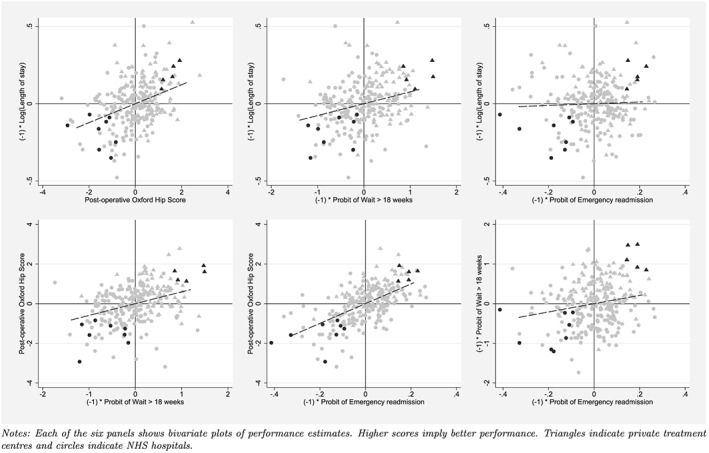
Multidimensional performance estimates

We now turn to the assessment of multidimensional hospital performance. Figure 3 shows the location of each hospital in the four‐dimensional performance space, where each panel presents scatter plots for two dimensions. For all performance dimensions, higher scores indicate better performance.

We identify five dominant and eight dominated hospitals at a probability level of 90% (highlighted dark grey). All dominant hospitals are private ISTCs that exclusively perform just planned orthopaedic procedures, here marked as triangles, whereas all dominated hospitals are public NHS hospitals, marked as circles, that provide a wider mix of services, including emergency care. 12Approximately 30% of admissions treated by consultants working in Trauma and Orthopaedics in NHS hospitals are classed as emergencies.Note, however, that not all ISTCs are located in NE quadrant and not all NHS hospitals are located in the SW quadrant. We re‐estimated the models and included an indicator variable for private ownership and found that, on average, ISTCs performed better on all dimensions. 13Length of stay (*β*=−0.100; *S* *E*=0.020; *p*<.001), post‐operative health status (*β*=1.205; *S* *E*=0.157; *p*<.001), probability of being readmitted (*β*=−0.084; *S* *E*=0.072; *p*<.001), and probability of waiting longer than 18 weeks (*β*=−0.820; *S* *E*=0.030; *p*=.007).Table 3 provides descriptive statistics for dominant and dominated providers in the financial year 2011/2012. Both groups are comparable in terms of the annual volume of NHS‐funded procedures provided. This suggests that volume–outcome effects may be less important in explaining overall performance differences. Conversely, we find that dominant providers operate in more competitive markets as indicated by the lower Herfindahl–Hirschman Index. This finding is consistent with the theory of quality competition in price‐regulated markets (Gaynor, Ho, & Town, 2015). Dominant providers may also be exploiting economies of scope: our specialisation index, which reflects the dispersion of HRGs treated within the orthopaedic department of hospital *j* and resembles a Gini index that is bounded between zero (= no specialisation) and one (= all patients of hospital *j* fall into one HRG; Daidone & D'Amico, 2009), suggests that good overall performance is associated with more concentrated service delivery. Note, however, that these comparisons are based on a small number of observations (*J*=13) and should be interpreted as associations.

**Table 3:** Characteristics of dominant and dominated providers (in 2011/2012)

	Dominant (*J*=5)			Dominated (*J*=8)	
Description	*M* *e* *a* *n*	*S* *D*		*M* *e* *a* *n*	*S* *D*
Annual volume of hip replacements	361.60	198.16		365.38	190.04
Ownership (1 = private, 0 = NHS)	1.00	‐		0.00	‐
Herfindahl–Hirschman Index	0.60	0.05		0.78	0.07
HRG specialisation	0.73	0.13		0.15	0.03

*Abbreviation:* NHS = National Health Service; HRG = healthcare resource group.

### 5.4 Comparison with approaches based on series of univariate probabilities

It is instructive to compare the results from our “full” MVMLM assessment with two alternative approaches: (a) a simple “univariate” approach and (b) an “intermediate” MVMLM regression that takes into account the correlation between performance during the estimation stage but treats performance estimates as independent. In both the simple and intermediate approaches, a hospital is judged to be dominant [dominated] if all four individual probabilities of exceeding [falling short of] the benchmarks are greater or equal to a specified probability threshold (“confidence box approach”). We adopt a Bonferroni correction to adjust for these multiple comparisons.

Table 4 shows the number of hospitals identified as dominant/dominated under each of these approaches. At a probability threshold of 90% (*P* *r* ^∗^=0.9), the univariate and intermediate MVMLM both identify just one or two dominant and dominated providers, which is fewer than the full MVMLM. The intermediate multivariate approach is generally more efficient than the univariate approach. The full MVMLM approach is most efficient and discriminatory at any probability threshold. The providers identified under each approach are always subsets of each other.

**Table 4:** Number of dominant/dominated providers under different estimation approaches and assumptions about the correlation between performance dimensions

Probability	(1) Univariate		(2) Intermediate multivariate		(3) Full multivariate	
threshold *P* *r* ^∗^	Dominant	Dominated	Dominant	Dominated	Dominant	Dominated
0.50	5	8	7	10	24	30
0.80	2	3	5	5	12	18
0.90	1	1	2	2	5	8
0.99	0	0	0	1	1	1

(1) Univariate approach—separate univariate models are estimated for each of the four performance dimensions.

(2) Intermediate multivariate approach—multivariate model is estimated, and correlation between performance dimensions is exploited in the estimation stage but ignored when forming probability statements.

(3) Fully multivariate approach—see Section 3.2 for details.

## 6 DISCUSSION

Rarely are stakeholders explicit about the valuations they attach to different dimensions of performance of organisations charged with serving the public, nor are these valuations likely to be identical. This makes it challenging to construct a composite performance indicator appropriate for all audiences. To circumvent this, we have set out a methodology for comparing providers in terms of their performance across a range of dimensions in a way that does not require valuation of each dimension and is consistent with economic theory. We extend previous literature by employing dominance criteria to compare providers against a multidimensional benchmark, and by constructing multivariate (rather than univariate) hypothesis tests of parameters that account for correlation between dimensions and thereby achieve correct coverage probabilities. Failure to perform multivariate tests can lead to incorrect inferences about multidimensional performance as we illustrate.

Dominance criteria have been adopted previously to assess ranking uncertainty related to the weights used in the construction of a composite indicator of performance (Schang et al. 2016), but our MVMLM offers two advantages over that study. First, Schang et al. do not account for uncertainty associated with the performance estimates themselves, whereas we are able to construct confidence statements for each performance dimension. Second, composite measures have been criticised because the aggregation of individual performance dimensions obscures where problems lie, thereby risking inaction by the organisations being evaluated (Smith, 2002). By reporting each performance dimension separately, the MVMLM approach offers greater transparency, indicating which dimension of performance should be the focus of attention for each organisation.

We apply our MVMLM approach to study the performance of English providers of hip replacement surgery with respect to four dimensions, namely, waiting time, length of stay, 28‐day emergency readmission, and patient‐reported health status after surgery. There are two main findings: First, performance is positively, albeit weakly, correlated across dimensions, which suggests that achievements on one dimension need not be traded off against those on another. We stress that these may not be causal estimates, although some of our findings confirm those of randomised controlled trials conducted in routine care settings. Second, all providers that dominate the benchmark are private ISTCs, whereas those dominated by the benchmark are public NHS hospitals. We do not believe that this is due to ISTCs treating easier cases, as we have controlled for a rich set of risk‐adjusters including, and unusually, pre‐treatment health status. This finding also accords with other studies which have found that ISTCs achieve better health outcomes than NHS hospitals (Browne et al., 2008; Chard, Kuczawski, Black, & van der Meulen, 2011) and discharge patients earlier (Siciliani, Sivey, & Street, 2013). As was hoped by those advocating the creation of treatment centres (House of Commons Health Committee, 2006), better performance may be the result of a more stream‐lined production process, with specialisation in treating elective joint replacement patients yielding performance advantages. The reasons for performance differences can rarely be ascertained definitively from routine data, but our analytical approach identifies the handful of organisations at both ends of the performance spectrum where deeper and more detailed investigation would be worthwhile.

The primary contribution of this work is methodological. We have laid out the rationale for and demonstrated the feasibility of using dominance criteria to judge hospital performance across multiple dimensions. The appeal of the dominance approach lies in the absence of strong assumptions about various stakeholders' utility functions and its ability to reduce multiple performance estimates into a single assessment. However, this comes at a price. Because the approach requires providers to perform better than the benchmark on all dimensions, there is no scope to compensate for average or poor performance on one dimension through excellent performance on another. Moreover, one would expect the number of providers identified as dominant or dominated to decrease as the number of dimensions under consideration increases (Pedraja‐Chaparro, Salinas‐Jimenez, & Smith, 1999). This is, of course, true for all multidimensional performance assessments—even those that weigh achievements—but the effect is more pronounced in a dominance framework with its stricter requirements.

Our framework can be extended to relax the requirements of strict dominance. Although regulators are unlikely to know the exact marginal rates of substitution for all stakeholders, they may be able to determine a reasonable range of likely values and rule out extreme cases. For example, it may be uncontroversial to assume that all stakeholders would be willing to accept a very small increase in waiting time (e.g., 0.5 days), in return for a large improvement in expected post‐operative health status (e.g., 8 points on the OHS). Our empirical framework can easily be extended to allow boundaries on the possible values of the marginal rates of substitution. Dominance criteria would then be applied within these boundaries.

A number of other extensions are possible. First, the statistical model can be extended to non‐normal (e.g., time‐to‐event and count) data by specifying generalised linear models or survival models. These would include in their linear predictor provider and/or patient level random effects that follow the same multivariate normal distribution as for the other outcomes (Gebregziabher et al., 2013; Teixeira‐Pinto & Normand, 2008; Verbeke, Fieuws, Molenberghs, & Davidian, 2014). Other multivariate distributions are possible as well, for example, the multivariate gamma. Note, however, that the added complexity of such models will often require the use of more flexible estimation techniques to evaluate the likelihood such as Markov chain Monte Carlo simulation.

Second, although shrinkage estimators are commonly applied in the context of performance assessment (Ash et al., 2012; Goldstein & Spiegelhalter, 1996), they have been criticised for being overly conservative (Austin, Alter, & Tu, 2003; Kalbfleisch & Wolfe, 2013). This is especially likely for providers with smaller case loads, with shrinkage moving them closer to the average and less likely to be identified as statistically significant positive or negative performers. Analysts may thus prefer to model provider effects using dummy variables, characterising uncertainty on the basis of the variance–covariance matrix.

Finally, multiple imputation techniques are a useful extension to the maximum likelihood framework when data on covariates are missing at random or to explore the impact of data missing not at random as part of sensitivity analyses (Carpenter & Kenward, 2013; Carpenter, Kenward, & White, 2007). In our study, non‐response with respect to the OHS is the most common reason for missing data, and other studies have shown that this is associated with patient characteristics and varies systematically across providers (Gutacker et al., 2015; Hutchings, Neuburger, Grosse Frie, Black, & van der Meulen, 2012; Hutchings, Neuburger, van der Meulen, & Black, 2014). Recently Gomes, Gutacker, Bojke, and Street (2016) used a multiple imputation approach to explore the effect of missing pre‐ or post‐operative OHS data on provider performance estimates within our dataset and found these to be robust but less efficient, that is, flagging fewer positive and negative performers as statistically significant. The estimates in this study, therefore, should be understood to be conservative.

In conclusion, in situations where the MRS are unknown or vary among stakeholders, the dominance approach provides a tractable means of evaluating multidimensional performance of public sector organisations.

## Supporting information

Table A1: Descriptive statistics for included and excluded observations
Table A2: Estimated coefficients and standard errors from multivariate regression model
Table A3: Estimated coefficients and stand ‐ ard errors‐multinomial hospital choice model (first‐stage)
Table A4: Correlation between performance dimensions ‐ exclud‐ing ISTCs
Table A5: Correlation between performance dimensions ‐ ac‐counting for provider average risk factors
