# Supplementary material for: Multidimensional performance assessment of public sector organisations using dominance criteria
Source: Health Econ. 2017 Aug 18;27(2):e13–27. doi: 10.1002/hec.3554 (PMC5900921; doi:10.1002/hec.3554)
Supplement: Supplementary file 1 — Table A1: Descriptive statistics for included and excluded observations Table A2: Estimated coefficients and standard errors from multivariate regression model Table A3: Estimated coefficients and stand ‐ ard errors‐multinomial hospital choice model (first‐stage) Table A4: Correlation between performance dimensions ‐ exclud‐ing ISTCs Table A5: Correlation between performance dimensions ‐ ac‐counting for provider average risk factors [file HEC-27-e13-s001.pdf]

Online appendix to 'Multidimensional performance assessment of public sector organisations using dominance criteria'

Table A1: Descriptive statistics for included and excluded observations

	Included			Excluded		
Description	N	Mean	SD	N	Mean	SD
Achievement measures (Dependent variables)						
Length of stay (in days)	95,878	5.34	3.64	90,017	6.05	4.37
Waiting time > 18 weeks	92,154	0.17	0.38	84,937	0.20	0.40
28-day emergency readmission	95,955	0.05	0.22	90,158	0.06	0.24
Patient characteristics (Control variables)						
Patient age (in years)	95,955	67.43	11.29	90,158	68.66	11.64
Patient gender (1=male, 0=female)	95,955	0.41	0.49	89,944	0.39	0.49
Primary diagnosis						
Osteoarthritis	95,955	0.06	0.24	90,158	0.08	0.28
Rheumatoid arthritis	95,955	0.93	0.25	90,158	0.91	0.29
Other	95,955	0.01	0.07	90,158	0.01	0.08
Number of Elixhauser comorbidities						
0	95,955	0.35	0.48	90,158	0.34	0.47
1	95,955	0.29	0.45	90,158	0.28	0.45
2-3	95,955	0.26	0.44	90,158	0.26	0.44
4+	95,955	0.10	0.31	90,158	0.13	0.33
Previously admitted as an emergency (1=yes, 0=no)	95,955	0.08	0.28	90,158	0.11	0.31
Socio-economic status	95,955	0.12	0.09	90,158	0.13	0.10
Healthcare Resource Group						
HB12C - category 2 without CC	95,955	0.77	0.42	90,158	0.75	0.43
HB11C - category 1 without CC	95,955	0.10	0.29	90,158	0.08	0.28
HB12B - category 2 with CC	95,955	0.07	0.26	90,158	0.07	0.26
HB12A - category 2 with major CC	95,955	0.04	0.19	90,158	0.04	0.21
HB11B - category 1 with CC	95,955	0.01	0.11	90,158	0.01	0.10
other	95,955	0.02	0.12	90,158	0.04	0.19

Legend: N = Number of observations, SD = Standard deviation; CC = complications or co-morbidities. Notes: Healthcare Resource Groups refer to major hip procedures for non-trauma patients in category 1 (HB12x) or category 2 (HB11x). Socio-economic status is approximated by the % of neighbourhood residents claiming income benefits. This characteristics is measured at neighbourhood level (lower super output area (LSOA)).

Table A2: Estimated coefficients and standard errors from multivariate regression model

	Length of stay		Post-operative OHS		Waiting time > 18 weeks		28-day emergency readmission	
Variable	Est	SE	Est	SE	Est	SE	Est	SE
Constant	2.078	0.052***	27.154	0.823***	-1.335	0.053***	-1.609	0.119***
FY 2010/11	-0.096	0.011***	0.043	0.072	0.114	0.045*	-0.008	0.019
FY 2011/12	-0.203	0.015***	0.229	0.085**	0.208	0.050***	-0.051	0.020*
Pre-operative OHS	-0.011	0.001***	0.599	0.016***			-0.005	0.001***
Pre-operative OHS^2	0.000	0.000***	-0.009	0.000***				
Patient age	-0.027	0.002***	0.208	0.025***			-0.014	0.004***
Patient age^2	0.000	0.000***	-0.002	0.000***			0.000	0.000***
Male patient	-0.074	0.004***	0.908	0.062***			0.142	0.015***
Primary diagnosis: Rheumatoid arthritis	0.026	0.022	-0.486	0.529			-0.086	0.113
Primary diagnosis: Other	0.035	0.009***	-1.169	0.187***			0.079	0.028**
Elixhauser comorbidities: 1	0.025	0.004***	-0.456	0.068***			0.061	0.017***
Elixhauser comorbidities: 2-3	0.068	0.004***	-1.433	0.083***			0.148	0.017***
Elixhauser comorbidities: 4+	0.153	0.007***	-2.826	0.133***			0.285	0.023***
Previously admitted as an emergency	0.071	0.005***	-0.613	0.124***			0.137	0.023***
Socio-economic status	0.003	0.001**	-0.523	0.027***			0.011	0.005*
Disabled	-0.036	0.003***	2.586	0.080***			-0.065	0.016***
Living alone	0.111	0.005***	-0.368	0.071***				
Symptom duration: 1 - 5 years	0.020	0.004***	-0.654	0.077***				
Symptom duration: 6 - 10 years	0.039	0.005***	-1.335	0.121***				
Symptom duration: > 10 years	0.055	0.007***	-1.712	0.159***				
Assistance in filling in PROM questionnaire	0.067	0.005***	-0.545	0.097***				
HRG: HB11C - category 1 without CC	0.037	0.006***						
HRG: HB12B - category 2 with CC	0.127	0.006***						
HRG: HB12A - category 2 with major CC	0.495	0.011***						
HRG: HB11B - category 1 with CC	0.122	0.016***						
HRG: other	0.376	0.031***						
First-stage residual	0.001	0.000	0.012	0.006*	0.001	0.001	0.003	0.001*
Var(θ_j)	0.025	0.002***	1.203	0.141***	0.378	0.038***	0.023	0.003***
Var(ϵ_{ij})	0.162	0.001***	68.563	0.340***	1.000		1.000	
Number of observations	95,955							

*** p< 0.001; ** p<0.01; * p<0.05

Legend: Est = Estimate; SE = Huber-White standard error (robust to unknown heteroscedasticity); OHS = Oxford Hip Score; HRG = Healthcare Resource Group; CC = Complications and comorbidities; FY = Financial year (April - March).

Notes: Socio-economic status is approximated by the % of neighbourhood residents claiming income benefits. This characteristic is measured at neighbourhood level (lower super output area (LSOA)).

Table A3: Estimated coefficients and standard errors - multinomial hospital choice model (first-stage)

Variable	Est	SE
Closest hospital	0.185	0.014***
Distance to hospital	-0.197	0.003***
Distance^2	0.001	0.0001***
Distance^3	-0.00002	0.000002***
Number of patients	95,955	
Number of providers	252	
Pseudo R^2	0.706	
$\chi^2(4)$	120,930	

*** p< 0.001; ** p<0.01; * p<0.05

Legend: Est = Estimate; SE = Huber-White standard error

Notes: Distance to hospital is measured as the straight-line distance from the centroid of the patient's lower super output area (LSOA) to the provider's headquarter (NHS trust) or hospital site (ISTCs). Distance is measured in kilometres.

Table A4: Correlation between performance dimensions - excluding ISTCs

Performance dimension	(1)	(2)	(3)	(4)
Length of stay (1)	1.00	***-0.13***	***0.02***	***0.02***
Post-operative OHS (2)	**-0.27**	1.00	***-0.02***	***-0.07***
Waiting time > 18 wks (3)	0.11	-0.02	1.00	*0.00*
28-day emergency readmission (4)	-0.03	**-0.46**	-0.02	1.00

Notes: Lower triangle reports the correlation between random effects at provider level, whereas upper triangle (in italics) reports the correlation between random effects (i.e. the idiosyncratic error term) at patient level. Bold indicates that the correlation is statistically significantly different from zero at the 95% level.

Table A5: Correlation between performance dimensions - accounting for provider average risk factors

Performance dimension	(1)	(2)	(3)	(4)
Length of stay (1)	1.00	***-0.13***	***0.02***	***0.02***
Post-operative OHS (2)	**-0.21**	1.00	***-0.02***	***-0.07***
Waiting time > 18 wks (3)	**0.19**	**-0.17**	1.00	*0.00*
28-day emergency readmission (4)	-0.08	**-0.35**	0.07	1.00

Notes: Lower triangle reports the correlation between random effects at provider level, whereas upper triangle (in italics) reports the correlation between random effects (i.e. the idiosyncratic error term) at patient level. Bold indicates that the correlation is statistically significantly different from zero at the 95% level.
